# Neurological and Mental Health Symptoms Associated with Post-COVID-19 Disability in a Sample of Patients Discharged from a COVID-19 Ward: A Secondary Analysis

**DOI:** 10.3390/ijerph19074242

**Published:** 2022-04-02

**Authors:** Martina Cacciatore, Alberto Raggi, Andrea Pilotto, Viviana Cristillo, Erika Guastafierro, Claudia Toppo, Francesca G. Magnani, Davide Sattin, Arianna Mariniello, Fabiola Silvaggi, Stefano Cotti Piccinelli, Nicola Zoppi, Giulio Bonzi, Stefano Gipponi, Ilenia Libri, Michela Bezzi, Paolo Martelletti, Matilde Leonardi, Alessandro Padovani

**Affiliations:** 1 Neurology, Public Health Disability Unit, Fondazione IRCCS Istituto Neurologico Carlo Besta, 20133 Milan, Italy; martina.cacciatore@istituto-besta.it (M.C.); erika.guastafierro@istituto-besta.it (E.G.); claudia.toppo@istituto-besta.it (C.T.); francesca.magnani@istituto-besta.it (F.G.M.); davide.satttin@istituto-besta.it (D.S.); arianna.mariniello@istituto-besta.it (A.M.); fabiola.silvaggi@istituto-besta.it (F.S.); matilde.leonardi@istituto-besta.it (M.L.); 2 Neurology Unit, Department of Clinical and Experimental Sciences, University of Brescia, 25121 Brescia, Italy; pilottoandreae@gmail.com (A.P.); viviana.cristillo@gmail.com (V.C.); stefanocottipiccinelli@gmail.com (S.C.P.); n.zoppi001@unibs.it (N.Z.); giuliobonzi85@gmail.com (G.B.); stebeat79@gmail.com (S.G.); ilenialibri92@gmail.com (I.L.); alessandro.padovani@unibs.it (A.P.); 3 Respiratory Unit, ASST Spedali Civili di Brescia, 25121 Brescia, Italy; michela.bezzi@asst-spedalicivili.it; 4 Department of Clinical and Molecular Medicine, Sapienza University, 00185 Rome, Italy; 5 Regional Referral Headache Center, Sant’Andrea University Hospital, 00189 Rome, Italy

**Keywords:** COVID-19, brain impairment, disability, cognitive dysfunction, anxiety, fatigue, hyposmia, hypogeusia, WHODAS-12

## Abstract

Recent studies suggest that COVID-19 survivors may experience long-term health consequences: in particular, neurological and mental health symptoms might be associated with long-term negative outcomes. This study is a secondary analysis of a larger cohort study and aims to determine the extent to which neurological and mental health sequelae are associated with survivors’ disability. Participants include COVID-19 survivors, with no pre-morbid brain conditions, who were discharged from the COVID-19 Unit of the ASST Spedali Civili Hospital between February and April 2020. At an average of 3.5 months after discharge, they were submitted to a neurological examination and completed the WHO Disability Assessment Schedule (WHODAS-12), the Hospital Anxiety and Depression Score, the Pittsburgh Sleep Quality Index and the Montreal Cognitive Assessment. Multivariable regression analysis was carried out to analyze variables that explain WHODAS-12 variation. In total, 83 patients (63 males, average age 66.9, 95% CI: 64.2–69.7) were enrolled; average WHODAS-12 was 13.2 (95% CI: 9.7–16.6). Cognitive dysfunction, anxiety, fatigue, and hyposmia/hypogeusia explained 28.8% of WHODAS-12 variation. These findings underline the importance and need for longitudinal follow-up assessments after recovery from COVID-19 and suggest the need for early rehabilitation of residual symptoms to enhance patients’ functioning.

## 1. Introduction

Since December 2019, coronavirus disease 2019 (COVID-19), caused by severe acute respiratory syndrome coronavirus 2 (SARS-CoV-2), has spread worldwide in a few months, causing a global pandemic [1]. Symptoms of COVID-19 vary according to the severity of the disease, from no symptoms to mild and moderate symptoms (e.g., cough, fever, muscle pain), up to more severe symptoms including pneumonia and respiratory failure [2], which can lead to death [3].

COVID-19 survivors may experience different health sequelae affecting several organ systems [4,5,6,7,8], including neurological manifestations such as fatigue, weakness, olfactory dysfunction, tremor and postural instability; cardio-respiratory long-term symptoms such as dyspnea and reduced exercise capacity; mental health disorders (especially in the medium- and long-term follow-up [9]) such as depression, anxiety, and sleep disturbances; and subtle cognitive impairment characterized by problems with concentration, memory, and praxes. Data from a meta-analysis showed that 63.2%, 71.9%, and 45.9% of patients experienced at least one persistent symptom at 30, 60, and 90 days after infection, respectively [10]. Moreover, the persistence of sequelae in survivors of COVID-19 has been highlighted by another meta-analysis at 7 months [11] and a year after infection [12]. Neurological and mental health symptoms, herein broadly defined as brain-related symptoms, have been suggested to constitute a relevant health problem in COVID-19 survivors and can potentially affect patients’ perceptions of their level of health, disability, and functioning.

Some studies have reported that COVID-19 survivors require post-acute rehabilitation and psychological support due to persisting sequelae and difficulties in daily life [13,14,15,16]. As a matter of fact, current data point out that COVID-19 survivors experience at least one limitation with daily life activities [17]. Among these limitations, difficulties with mobility (such as walking, climbing stairs, or lifting weights [17,18,19]) and limitations in living alone without assistance [20], as well as in returning to daily social and work activities [17,20,21], were observed. However, the real impact of brain-related sequelae of COVID-19 on disability levels has not been systematically addressed. Therefore, the main possible target of post-acute neurological rehabilitation and support has not been identified.

We moved from the hypothesis that brain-related symptoms—which are commonly found in COVID-19 survivors—might reasonably predict disability outcome. The extent to which disability variation can be predicted by such symptoms is, at present, not systematically evaluated, but is of relevance in the planning of post-acute treatment. Therefore, the aim of this manuscript is to address the impact of a set of new onset neurological and mental health symptoms on post-COVID-19 disability. To pursue this objective, we addressed how much disability variation is explained by brain-related symptoms in a group of COVID-19 survivors without a history of neurological and mental health diseases before SARS-CoV-2 infection.

## 2. Materials and Methods

The present study was designed according to the ethical standards of the Declaration of Helsinki and received approval from the local ethics committee of ASST “Spedali Civili di Brescia” Hospital; the requirement for informed consent was waived by the Ethics Commission (NP 4166).

### 2.1. Participants

This study is a secondary analysis of the NEXT study, a larger cohort study aimed to describe the health status and the impact of neurological and mental health symptoms of COVID-19 survivors with no pre-COVID-19 history of neurological and mental health diseases [22,23]. 

A total of 105 patients were enrolled in the NEXT study, and included those who survived COVID-19 and were discharged from the COVID-19 Unit of the ASST Spedali Civili Hospital between February and April 2020. Patients with premorbid psychiatric and neurological conditions were excluded. Within six months after discharge, a full neurological examination (which included a neurological symptoms’ checklist) was planned. The time of the follow-up was variable in consideration of patients’ clinical status and pandemic situation.

The patients included in this study were those who, in addition to the neurological examination, also completed the 12-item WHO Disability Assessment Schedule (WHODAS-12) [24], herein used as primary outcome.

### 2.2. Assessment

The WHODAS-12 is a short version of the 36-item WHODAS 2.0, and it includes items from each of the six disability domains (namely understanding and communicating, getting around, self-care, getting along with people, household activities, work activities, and participation in society) [24]. The questionnaire score range is 0–100, with higher scores reflecting higher disability level, and it explains 81% of the variance of the 36-item version. The protocol also included the Hospital Anxiety and Depression Scale (HADS) [25], the Pittsburgh Sleep Quality Index (PSQI) [26], and the Montreal Cognitive Assessment (MoCA) [27]. The HADS is a 14-item questionnaire addressing symptoms of depression and anxiety, and each subscale is composed of seven items: scores range between 0 and 21, with higher scores reflecting more severe symptoms, and a score ≥ 8 identifies relevant anxiety or depression. The PSQI is a 19-item questionnaire that addresses seven sleep-related factors (subjective sleep quality, sleep latency, sleep duration, sleep efficiency, sleep disturbances, use of sleep medication, and daytime dysfunction), each of which is scored on a 0–3 scale: the total score thus ranges from 0 to 21, and a score ≥ 5 identifies poor sleep. The MoCA is a short assessment of cognitive functioning, which includes tests for short-term and delayed verbal memory, visuo-spatial abilities, executive function, attention, concentration, working memory, language, and orientation to time and space. The total score ranges between 0–30 and a score ≤ 25 indicates cognitive dysfunction.

Variables of interest to address disability variation were brain-related symptoms, which were rated in terms of their presence or absence using a specific symptom checklist. Neurological symptoms—namely confusion, dizziness, postural instability, gait disturbances, fatigue, numbness/tingling, blurred vision or vision loss, hyposmia/hypogeusia, urinary dysfunction, swallowing difficulties, headaches, and myalgia—were evaluated using a structured form [22]. Mental health symptoms, evaluated through the questionnaires used in the protocol, included cognitive dysfunction (MoCA ≤ 25), anxiety and depression (HADS—anxiety and HADS—depression ≥ 8), and sleep disturbances (PSQI ≥5). 

Hospitalization data included the Cumulative Illness Rating Scale (CIRS) [28] and COVID-19 severity, classified according to the Brescia-COVID Respiratory Severity Scale (BCRSS) [29]. The CIRS is a 14-item scale addressing presence and severity of comorbidities: each item represents possible organs affected by a chronic disease, and it is scored from 0 to 4. The CIRS provides two scores: the Comorbidity Score, which is the sum of all scores assigned to 14 items, and the Severity Index, consisting of the number of items ranking three or four in disease severity. The BCRSS is a 0–3 score classification of COVID-19 severity of non-intubated patients, which relies on four criteria: (1) dyspnea or staccato speech, defined as being unable to count rapidly up to 20 after a deep breath, at rest, or during minimal activity; (2) respiratory rate > 22 breaths/min; (3) PaO2 < 65 mmHg or SpO2 < 90% with supplemental oxygen; and (4) relevant worsening of chest radiograph.

### 2.3. Data Analysis

Continuous variables were described using means and 95% confidence intervals (95% CI), categorical variables with frequencies and percentages. Information on hospitalization, CIRS, and BCRSS were used for descriptive purposes.

The associations between the variables addressing brain-related symptoms were assessed using Kendall’s TAU correlation coefficient (strong if TAU >0.500). Each variable was then tested for its ability to explain WHODAS-12 score variation in a univariable regression model: those that significantly explained WHODAS-12 variation were retained for a multivariable model. Retained variables were entered together and excluded with a backward procedure, which excluded variables one at time. At each step, the one with the lowest predictive power was excluded and we set *p* < 0.05 as a criterion for retention: in this way, the final model will include only significant predictors. For each predictor, partial correlation (i.e., the relation between each predictor and the outcome variable, controlling for the effects of all other predictors) and part correlation (i.e., the relation between each predictor and the outcome, controlling for the effect that the other predictors have on the outcome and which represents the unique relation between each single predictor and the outcome) were also calculated. Data were analyzed using IBM SPSS statistics (v. 27.0).

## 3. Results

Out of the 105 patients discharged between February and April 2020, the full protocol was available for 83. Of them, 63 were males (75.9%), and the average age was 66.9 (95% CI: 64.2–69.7). Hospitalization lasted, on average, 13 days (95% CI: 10.7–15.2), and 66 patients (79.5%) received oxygen therapy (1 was intubated and 10 received non-invasive ventilation) for an average of 8.2 days (95% CI: 6.4–10.0). Average total CIRS score at hospitalization was 23.6 (95% CI: 22.7–24.5) and average CIRS severity was 1.82 (95% CI: 1.74–1.89); 37 patients scored 1 on the BCRSS (44.6%), 12 scored 2 (14.5%), and the remaining patients scored 0. The average elapsed time between discharge and follow-up was 3.5 months (100 days; 95% CI: 95–104). Mean and 95% CI scores were 24.1 (23.4–24.8) at MoCA; 6.5 (5.9–7.2) at PSQI; 4.0 (3.3–4.7) at HADS—anxiety and 3.6 (2.9–4.4) at HADS—depression; 13.2 (9.7–16.6) at WHODAS-12.

Kendall’s TAU correlation (see Table 1) showed that no coefficient was higher than 0.500, the strongest associations being between fatigue and gait disturbances (TAU = 0.440), fatigue and myalgia (TAU = 0.428), headache and dizziness (TAU = 0.427), all with, *p* < 0.001.

Table 2 shows the prevalence of brain-related symptoms and the regression models. The most common symptoms at follow-up were sleep disturbances, cognitive dysfunctions, and fatigue. The results of the regression model show that cognitive dysfunction, anxiety, fatigue, and hyposmia/hypogeusia explained 28.8% of WHODAS-12 variation. Partial and part correlation suggest that the largest relations between brain-related symptoms and disability outcomes were observed for hyposmia/hypogeusia and cognitive dysfunction.

## 4. Discussion

The results show that cognitive dysfunction, anxiety, fatigue, and hyposmia/hypogeusia, present in 61.4%, 14.5%, 30.1%, and 18.1% of a group of COVID-19 survivors evaluated at 3.5 months from hospital discharge, explained 28.8% of WHODAS-12 variation. The presence of long-term brain-related symptoms, thus, is a potential driver of increased disability in COVID-19 survivors.

Previous studies have shown that COVID-19 survivors, in addition to long-term symptoms, report limitations in daily life [30,31,32]. Specifically, age, male sex, mechanical ventilation, and length of stay in intensive care units were associated with reduced quality of life (QoL) and functioning, with 36% of COVID-19 survivors reporting persistent functional limitations [30] (Grades 2 and 4 of the post-COVID-19 Functional Status scale [33]). Furthermore, neurocognitive deficits and psychiatric symptoms, such as executive function deficits, anxiety, and depression, have been associated with reduced physical and mental aspects of QoL [32]. 

Our study provides evidence on brain-related symptoms as potential drivers of disability, which should therefore be the target of multidisciplinary rehabilitative interventions in COVID-19 survivors [34,35]. The rehabilitation and treatment of fatigue, symptoms of anxiety, cognitive dysfunction, and hyposmia/hypogeusia—previously reported by between 36% [31] and 87% [36] of COVID-19 survivors—might therefore impact on approximately one-third of patient-reported disability among patients who were discharged from a COVID-19 ward. Addressing this disability is of great relevance, as it is likely associated with long-COVID syndrome (LCS), a condition that can develop after recovery from COVID-19. Although the definition of LCS is still debated, there is an agreement on acknowledging the presence of COVID-19-related symptoms (including, but not limited to, fatigue, shortness of breath, and cognitive dysfunction, with an impact on everyday functioning) for more than 3 months after the diagnosis of SARS-CoV-2 infection or symptoms’ onset [37]. It is quite difficult to estimate the prevalence of LCS in the population; however, in light of the dramatic increase in cases of COVID-19 infection, due to the so-called fourth wave driven by the Omicron variant, it is reasonable to assume that a large number of people could suffer from this condition in the future, stressing the need to identify and promptly treat the potential brain-related sequelae of COVID-19. 

Longitudinal cognitive evaluations are recommended in COVID-19 survivors to promptly detect potential disorders and provide tailored interventions [38,39]. Highlighting and treating the cognitive sequelae in COVID-19 survivors is important to limit the impacts on the mental, physical, and cognitive well-being of those who have recovered from COVID-19. Subjective memory deficits (which are common after COVID-19 [40]) are associated with an increased risk of future mild cognitive deterioration or dementia, especially in the elderly [41]. In addition to this, conditions such as anxiety and depression have a strong impact on increased disability level in general populations [42] and have been shown to influence quality of life of COVID-19 survivors [32]. The results of our study expand the evidence available for quality of life to include disability outcome. In fact, both anxiety and depression impact on WHODAS-12 variation in univariable models, and anxiety was also found to have independent predictive power on our final multivariable model. Cognitive functioning (e.g., memory and executive function) is also strongly associated with different neurological symptoms associated with COVID-19, such as headache or anosmia, and the presence of depression and anxiety was also associated with the presence of cognitive complaints [43]. It must also be noted that olfactory and cognitive dysfunction are associated with COVID-19 survivors, as we have already shown in a previous publication derived from the entire NEXT sample [23]. Olfactory dysfunction is common in normal ageing, but it is also associated with age-related neurodegenerative conditions [44] and the presence of cognitive deficits [45], thus suggesting that patients with such residual symptoms are to be considered particularly vulnerable to the negative long-terms effects of SARS-CoV-2 infection. The fact that these two brain-related symptoms were those showing the largest part and partial correlation, i.e., the largest unique association with the disability outcome, expands the previous observation on their association [23,44,45], thereby making it relevant to address them in order to positively impact on patients’ disability.

Evidence from the available literature, together with the results of our study, stress the need for the early assessment of brain-related sequelae of COVID-19 to reduce patients’ level of disability. Multidisciplinary interventions should therefore include physical rehabilitation activities with programs to improve strength and fatigue [46,47], as well as psychological interventions with mental health assessment, pharmacological treatments, individual or group psychotherapies [48], or online self-management programs [49]. Future studies should be planned to implement and validate tailored rehabilitation interventions, taking into account the consequences of COVID-19 as a whole. The pandemic offers the possibility to re-think how health and social services are planned: in particular, it provides the opportunity to invest in prevention and health promotion [50].

Our study presents some limitations. First, it is a monocentric study with a small number of subjects; therefore, caution is warranted in the generalization of our results. Second, two cases presented as outliers of the WHODAS-12; we decided not to delete or replace them with central values to avoid a reduction in sample size or having a replacement with a value that is clearly different from those reported by the two cases. The presence of three outliers, however, might have influenced the result of the linear regression. Third, the cross-sectional feature of our analyses prevented us from drawing strict causal relationships. A within-subjects assessment for disability measures would have allowed us to determine whether any change in patient-reported disability could be observed. Future studies with larger sample sizes and a longitudinal design should be carried out to confirm our results.

## 5. Conclusions

In sum, we found that that cognitive dysfunction, anxiety, fatigue, and hyposmia/hypogeusia explain almost one-third of COVID-19 survivors’ disability level, measured with WHODAS-12. These findings stress the need for longitudinal follow-up assessments after recovery from COVID-19 and suggest the need for early rehabilitation of patients who develop LCS.

## Figures and Tables

**Table 1:** Kendall’s TAU correlation between the variables.

	1	2	3	4	5	6	7	8	9	10	11	12	13	14	15	16
1. Cognition	--															
2. Anxiety	−0.026	--														
3. Depression	0.273 *	0.200	--													
4. Sleep	0.108	0.239 *	0.098	--												
5. Confusion	0.255 *	0.318 **	0.106	0.160	--											
6. Dizziness	0.092	−0.002	0.071	0.114	0.181	--										
7. Postural instability	0.240 *	0.122	0.346 **	0.077	0.368 **	0.326 **	--									
8. Gait disturbances	0.237 *	0.243 *	0.027	0.146	0.243 *	0.204	0.393 **	--								
9. Fatigue	0.196	0.178	0.295 **	0.080	0.178	0.195	0.273 *	0.440 **	--							
10. Numbness/tingling	0.062	0.234 *	0.130	0.211	0.234 *	0.034	0.023	0.181	0.222 *	--						
11. Vision blurred loss	0.262 *	0.146	0.378 **	0.074	0.146	0.269 *	0.291 **	0.259 *	0.345 **	0.348 **	--					
12. Hyposmia/hypogeusia	0.115	0.252 *	0.142	0.201	0.163	0.290 **	0.421 **	0.278 *	0.169	0.159	0.009	--				
13. Urinary dysfunction	0.225 *	−0.002	0.160	0.188	0.272 *	0.227 *	0.326 **	0.393 **	0.265 *	0.330 **	0.351 **	0.123	--			
14. Swallowing difficulties	0.097	−0.104	0.030	−00.86	0.184	0.156	0.105	0.200	0.165	0.086	0.261 *	0.013	0.292 **	--		
15. Headache	−0.008	0.040	00.30	00.31	0.184	0.427 **	0.105	0.200	0.275 *	0.086	0.133	0.144	−0.114	−0.064	--	
16. Myalgia	0.099	0.089	0.145	0.134	0.169	0.348 **	0.133	0.361 **	0.428 **	0.320 **	0.439 **	0.028	0.348 **	0.213	0.094	--

Note. * correlation is significant at *p* < 0.05 level; ** correlation is significant at *p* < 0.01. All coefficients are 2-tailed. Numbers in the first row represent variables as indicated in the first column.

**Table 2:** Prevalence of brain-related symptoms among COVID-19 survivors and regression model predicting WHODAS-12 variation.

	N (%) of Patients Reporting Each Symptom	Univariable Linear Regression	Multivariable Linear Regression: Initial Model		Multivariable Linear Regression: Final Model	
		B (SE) *p*-Value	B (SE) *p*-Value	Partial Correlations Part Correlations	B (SE) *p*-Value	Partial Correlations Part Correlations
Hyposmia/Hypogeusia	15 (18.1%)	17.6 (4.4) *p* < 0.001	10.6 (4.7) *p* = 0.026	0.254 0.214	12.8 (4.3) *p* = 0.004	0.321 0.280
Anxiety	12 (14.5%)	14.4 (5.1) *p* = 0.006	9.0 (4.9) *p* = 0.070	0.207 0.173	9.4 (4.7) *p* = 0.049	0.221 0.188
Cognitive dysfunction	51 (61.4%)	10.1 (3.6) *p* = 0.007	6.4 (3.5) *p* = 0.072	0.206 0.172	7.7 (3.3) *p* = 0.024	0.253 0.217
Fatigue	25 (30.1%)	12.3 (3.8) *p* = 0.002	6.4 (4.0) *p* = 0.116	0.181 0.150	7.6 (3.6) *p* = 0.036	0.235 0.200
Depression	13 (15.7%)	13.6 (4.9) *p* = 0.007	3.3 (5.1) *p* = 0.523	0.074 0.060	–	–
Postural instability	7 (8.4%)	22.5 (6.2) *p* = 0.001	8.3 (7.1) *p* = 0.243	0.135 0.111	–	–
Gait disturbances	11 (13.3%)	13.5 (5.3) *p* = 0.013	−0.9 (5.9) *p* = 0.874	−0.018 −0.015	–	–
Sleep disturbances	62 (74.7%)	7.3 (4.2) *p* = 0.088	–	–	–	–
Confusion	12 (14.5%)	8.9 (5.2) *p* = 0.090	–	–	–	–
Dizziness	14 (16.9%)	1.2 (5.0) *p* = 0.810	–	–	–	–
Numbness/Tingling	21 (25.3%)	0.1 (4.3) *p* = 0.974	–	–	–	–
Blurred vision or vision loss	16 (19.3%)	3.7 (4.7) *p* = 0.446	–	–	–	–
Urinary dysfunction	14 (16.9%)	5.0 (5.0) *p* = 0.320	–	–	–	–
Swallowing difficulties	5 (6.0%)	−1.5 (7.8) *p* = 0.851	–	–	–	–
Headaches	5 (6.0%)	5.6 (7.8) *p* = 0.474	–	–	–	–
Myalgia	20 (24.1%)	−0.8 (4.4) *p* = 0.848	–	–	–	–
R			0.580		0.580	
R^2^ [Adjusted R^2^]			0.336 [0.274]		0.314 [0.288]	
Regression mean square			1122.7		1834.2	
Residual mean square			207.1		205.8	
F (*p*-value)			5.42 (<0.001)		8.91 (<0.001)	

## Data Availability

Data available in an accessible repository on request.
